# Increasing Effectiveness of Cognitive Behavioral Therapy for Conduct Problems in Children and Adolescents: What Can We Learn from Neuroimaging Studies?

**DOI:** 10.1007/s10567-021-00346-4

**Published:** 2021-03-08

**Authors:** Walter Matthys, Dennis J. L. G. Schutter

**Affiliations:** 1 Department of Child and Adolescent Studies, Utrecht University, Heidelberglaan 1, P.O. Box 80140, 3584 CS Utrecht, The Netherlands; 2 Department of Psychiatry, University Medical Center Utrecht, Heidelberglaan 100, P.O. Box 85500, 3508 GA Utrecht, The Netherlands; 3 Department of Experimental Psychology, Helmholtz Institute, Utrecht University, Heidelberglaan 1, 3584 CS Utrecht, The Netherlands

**Keywords:** Cognitive behavioral therapy, Conduct problems, Neuroimaging, Children, Adolescents, Residential treatment

## Abstract

Cognitive behavioral therapy (CBT) is particularly relevant for children from 7 years on and adolescents with clinical levels of conduct problems. CBT provides these children and adolescents with anger regulation and social problem-solving skills that enable them to behave in more independent and situation appropriate ways. Typically, CBT is combined with another psychological treatment such as behavioral parent training in childhood or an intervention targeting multiple systems in adolescence. The effectiveness of CBT, however, is in the small to medium range. The aim of this review is to describe how the effectiveness of CBT may be improved by paying more attention to a series of psychological functions that have been shown to be impaired in neuroimaging studies: (1) anger recognition, (2) the ability to generate situation appropriate solutions to social problems, (3) reinforcement-based decision making, (4) response inhibition, and (5) affective empathy. It is suggested that children and adolescents first become familiar with these psychological functions during group CBT sessions. In individual sessions in which the parents (and/or child care workers in day treatment and residential treatment) and the child or adolescent participate, parents then learn to elicit, support, and reinforce their child’s use of these psychological functions in everyday life (in vivo practice). In these individual sessions, working on the psychological functions is tailored to the individual child’s characteristic impairments of these functions. CBT therapists may also share crucial social-learning topics with teachers with a view to creating learning opportunities for children and adolescents at school.

## Introduction

According to the most recent large meta-analysis of psychological therapy for children and adolescents treated for mental health problems in the clinical range the mean post-treatment effect size (ES, Cohen’s *d*) for conduct problems is 0.46 (Weisz et al., 2017). The effect sizes of several types of psychological therapy for conduct problems, however, differ from each other.

In the meta-analysis by McCart et al. (2006), the ES of cognitive behavior therapy (CBT) in children and adolescents with conduct problems is *d* = 0.35 while the ES of behavioral parent training is *d* = 0.47. According to a more recent meta-analysis of behavioral parent training, the ES for children aged 2–9 years is even larger: *d* = 0.69 (Leijten et al., 2019). In the meta-analysis by McCart et al., there was a positive relationship between age and ES for CBT: as youth enter more advanced levels of cognitive development, they receive increased benefits from CBT. Yet in the meta-analysis by Armelius and Andreassen (2007), the ES of CBT in youths aged 12–22 for the treatment of antisocial behavior in residential setting is only *d* = 0.25. According to British guidelines, group CBT for children aged 9–14 years with clinical levels of conduct problems is advised based on low-to moderate-quality evidence from randomized controlled trials (National Institute for Health and Clinical Practice (NICE), 2013, 2017; Pilling et al., 2013). Clearly, attempts at increasing effectiveness of CBT as a psychological treatment for conduct problems in middle childhood and adolescence are appropriate.

One way might be to examine neuroimaging studies that investigate biological correlates of psychological functions targeted in CBT. In CBT, children and adolescents learn better ways to manage their anger and solve social problems by increasing emotion-regulation and problem-solving abilities (Lochman et al., 2019; Matthys & Lochman, 2017). In particular, children and adolescents learn to identify their level of anger as well as to use coping self-statements, distraction techniques, and brief deep-breathing relaxation methods as a means to handle arousal associated with their anger. They also learn and improve to adequately interpret social problems, generate possible solutions, and decide which solution will be implemented. We will currently review functional magnetic resonance imaging (fMRI) studies examining psychological functions that are targeted in CBT for conduct problems: anger recognition, the ability to generate situation appropriate solutions to social problems, reinforcement-based decision making, response inhibition, and affective empathy. For this, we use meta-analyses, reviews of neuroimaging studies and separate neuroimaging studies, and discuss results in the context of other studies of these five psychological functions. Thus, our non-systematic review of functional neuroimaging studies is motivated by increasing our understanding of psychological abilities that may improve CBT effectiveness. After presentation of the neuroimaging work of each psychological function, we will provide a conclusion as a theoretical statement and working hypothesis about the potential role of the psychological function in the maintenance of conduct problems and finally discuss possible implications for CBT.

## Cognitive Behavioral Therapy

CBT is particularly relevant for children from 7 years on and for adolescents as CBT provides them with anger regulation and social problem-solving skills that enable them to behave in more independent and situation appropriate ways. Anger management and social problem solving are core elements of evidence-based practice for children with conduct problems (Garland et al., 2008). Early CBT programs such as the Anger Control Program (Lochman et al., 1981) and Problem-Solving Skills Training (Kazdin et al., 1987) were developed as sole interventions for children with conduct problems. For example, Problem-Solving Skills Training was offered in cases when working with parents was not a viable option due to severe family dysfunction or parent psychopathology (Kazdin et al., 1987).

Over the years, developers of programs have combined CBT with other psychological treatments such as behavioral parent training in childhood or intervention targeting multiple systems in adolescence. Examples of programs for children aged 7–13 years that have been proven to be effective are Problem-Solving Skills Training combined with Parent Management Training (Kazdin et al., 1992); the Coping Power program, a more extended and comprehensive version of the Anger Coping program (Lochman et al., 1981), consisting of a child component and a parent component (Lochman et al., 2008; Wells et al., 2008; Van de Wiel et al., 2007; Zonnevylle-Bender et al., 2007); the Stop Now and Plan program consisting of several components including a child component and a parent component (Augimeri et al., 2007; Burke & Loeber, 2015). An example of a program developed for adolescents is the Aggression Replacement Training (Goldstein et al., 1998; Hornsveld et al., 2015) which involves network meetings between parents, teachers, friends, and social workers or other care providers. For adolescents with severe conduct problems interventions that target multiple environmental systems (e.g., family, peers, school) such as Multisystemic Therapy (Henggeler et al., 2009; Van der Stouwe et al., 2014) have been developed and may also include a CBT component. For more examples of evidence-based programs, see reviews by Kaminski and Claussen (2017) and McCart and Sheidow (2016).

Most psychological interventions for conduct problems that have been proven to be effective are based on operant learning principles (e.g., positive reinforcement) and cognitive learning principles (e.g., use of inner speech). In behavioral parent training, children and adolescents acquire appropriate behaviors and learn to refrain from inappropriate behaviors as a result of parents’ or caregivers’ giving positive instructions, praising appropriate behaviors, ignoring minor inappropriate behaviors, and using time-out for severe inappropriate behaviors (Kazdin, 2005). Likewise, in CBT, children and adolescents acquire anger management and problem-solving abilities (Lochman et al., 2019; Matthys & Lochman, 2017). The effectiveness of these social-learning-based behavioral therapies may depend on children’s and adolescents’ impairments in processing of punishment cues, reward cues, and cognitive control (Matthys et al., 2012, 2013).

In the present review, we will focus on conduct problems in the clinical range, either because children and adolescents meet criteria of Oppositional Defiant Disorder (ODD) or Conduct Disorder (CD) according to the *Diagnostic and Statistical Manual of Mental Disorders* (fifth edition; *DSM-5*, American Psychiatric Association, 2013), Oppositional Defiant Disorder or Conduct-Dissocial Disorder according to the *International Classification of Diseases* (eleventh edition; *ICD-11*, World Health Association, 2020), or because they show symptoms in the clinical range on a standardized measure of psychopathology. Finding ways to improve the effectiveness of psychological treatment of conduct problems is of utmost importance, especially given the range of short- and long-term negative outcomes of conduct problems in adulthood, including crime, substance use disorders, suicide attempts, low educational achievement, anxiety disorders, depressive disorders, manic episode, schizophreniform disorder, eating disorders (Fergusson et al., 2005; Kim-Cohen et al., 2003), as well as high costs in terms of service utilization across all three domains of criminal justice, health, and social welfare (Rivenbark et al., 2018).

## Anger Recognition

Emotional dysregulation during anger is an important mechanism driving reactive aggression in children and adolescents (Hubbard et al., 2010). Based on clinical work with low-income aggressive children, Lochman et al. (1981) developed the Anger Control program that incorporated both the self-instruction training methods from Meichenbaum (1977) and the social problem-solving training methods from Spivack and Shure (1974). Anger management skills are crucial for children and adolescents with conduct problems in order to handle the surge in anger to a provocation or frustration before they can successfully begin to use problem-solving strategies. Therefore, in the Coping Power program (Lochman et al., 2008), anger coping precedes social problem solving. However, a precondition for children and adolescents with conduct problems to learn managing their anger is to become aware and recognize their own anger.

It might be that children and adolescents with conduct problems have difficulties recognizing anger in others and their own anger. These difficulties have been associated with hyporeactivity of the orbitofrontal and anterior cingulate cortex involved in the processing of angry expressions (Blair et al., 1999). Male adolescents and young adults with conduct problems displayed abnormally reduced brain responses of the amygdala, ventromedial prefrontal cortex, orbitofrontal cortex, and insula when viewing angry versus neutral faces relative to controls (Passamonti et al., 2010). Also, female adolescents with conduct problems demonstrated decreased medial orbitofrontal cortex functioning while viewing facial expressions among which anger relative to controls (Fairchild et al., 2014). In addition, reduced left anterior insula and inferior frontal gyrus responses have been observed in male adolescents with conduct problems and callous-unemotional (CU) traits relative to normal controls when participants were asked to judge their *own* emotional reactions to fearful and angry expressions of others (Klapwijk et al., 2016).

Several neuroimaging studies suggest that children and adolescents with conduct problems have difficulty in recognizing anger in others and their own feelings of anger. An efficacy study can test the hypothesis whether including anger recognition as a crucial first step in anger management improves anger management abilities and reduces reactive aggression, especially in those children and adolescents with clear difficulties in anger recognition.

In CBT, improving children’s and adolescents’ anger management abilities is an important topic. For example, in the Coping Power program, one session is devoted to identification of physiological cues of anger (e.g., feeling hot, faster heart rate, tightened muscles) and identification of various levels of anger (e.g., irritated, mad, furious) (Lochman et al., 2008). One may question whether one session is sufficient for children with conduct problems to recognize their anger in everyday life situations as this seems to be a major problem for them. In Coping Power trials, the effect sizes for proactive aggression have been as much as three times larger than the effect sizes for reactive aggression (Miller et al., 2020). From the viewpoint of enhancing the effects of the program by more precisely and intensively targeting the active mechanisms of reactive aggression, developers of Coping Power will start to include mindfulness in the program (Miller et al., 2020). But in their motivation the authors do not seem to consider anger recognition problematic for children and adolescents with conduct problems as no reference is made to psychological studies showing that anger recognition may be problematic for children and adolescents with conduct problems.

Male young offenders demonstrate difficulties in recognizing low intensity anger in others (Bowen et al., 2016). Likewise, boys and girls with disruptive behavior referred into a crime prevention program were impaired in anger recognition (Hunnikin et al., 2020). Anger recognition was disproportionally impaired in boys with early-onset CD (Fairchild et al., 2009). Also, both boys and girls with conduct problems compared to controls showed more difficulties in recognizing facial emotions among which anger (Kohls et al., 2020). In their study of social information-processing in aggressive and depressed children, Quiggle et al. (1992) included the emotions (e.g., anger, sadness) when children were read negative stories. Depressed children reported more inner experienced anger than controls, but surprisingly aggressive children did not. Likewise, a study by Van Rest et al. (2020) in adolescents with conduct problems did not show differences between these adolescents and controls in their anger after viewing videos depicting problem situations in which youths were disadvantaged by accident, while these adolescents generated more aggressive responses and selected more often an aggressive response among various responses shown. Apparently, these studies have been overlooked by researchers involved in CBT but results are in line with the previously discussed neuroimaging studies. How can anger recognition become a topic in CBT?

In CBT, child and parent components often are offered as separate components: therapists work with parents on their parenting skills and with children on their anger management and social problem-solving skills. Here, we propose that parents or foster parents should become actively involved in CBT; this also applies for child care workers in day treatment and residential treatment. For example, parents can observe their child and therapist working on anger recognition, including the recognition of physiological cues which signal that the child is becoming angry. Parents then learn to develop skills in prompting and praising their child’s use of anger recognition in everyday life (in vivo practice, Kazdin et al., 1989). For example, they learn to ask questions such as ‘It seems to me that something is bothering you’ or ‘It seems to me that you feel annoyed,’ and ‘Good of you that you recognize this in yourself.’ In our view, anger recognition in everyday life should become a major theme in CBT as it is a precondition for the next steps consisting of using the coping self-statements, distraction techniques, and brief deep-breathing relaxation methods as a means to handle arousal associated with anger by the child or adolescent.

Furthermore, CBT therapists may also want to inform teachers of the child’s or adolescent’s learning processes. The school is an ideal environment for children and adolescents with conduct problems to improve their anger management and social problem-solving skills given the amount of time they spend in school in a wide variety of social contexts. With regard to anger recognition, we assume that a lot of practice is needed for children and adolescents with conduct problems to actually recognize their anger in socially difficult situations. Like the parents, teachers may prompt and praise the child’s use of anger recognition in everyday life. In addition, teachers may warn the child that a difficult situation is coming for him or her. For example, the child quickly becomes angry when he receives a comment about his school work. The anticipation of a difficult situation may help the child to better recognize his anger and use the anger management skills he or she learns in CBT. Other potential difficult situations are when the child is teased by peers or when a peer outperforms in a game (Dodge et al., 1985). These are all problematic social situations that will be addressed in the next section.

## The Ability to Generate Situation Appropriate Solutions to Social Problems

In everyday life, children continuously face social problems such as how to respond to situations in which the child is being disadvantaged or how to cope with competition (Dodge et al., 1985; Matthys et al., 2001). To deal with these challenges, children have at their disposal a set of cognitive skills including defining the problem or interpreting the situation, generating possible solutions, and deciding which solution will be implemented (see social information-processing models by Crick & Dodge, 1994, and by Dodge et al., 1986). In social information-processing research emphasis is put on the aggressive children’s interpretation of the situation as being hostile (see meta-analyses by De Castro et al., 2002, and by Verhoef et al., 2019). On the other hand, only few studies have focused on the quantity and the quality of the solutions generated. As for the number of solutions, boys with conduct problems have been found to generate fewer solutions to problems than their peers in situations in which they have to cope with competition (Matthys et al., 1999). With respect to the quality of the responses generated, aggressive children have been shown to offer fewer verbal assertive solutions than their peers (Lochman & Lampron, 1986). For treatment purposes, the latter finding is highly relevant: do children and adolescents with conduct problems have appropriate responses in their repertoire?

When individuals demonstrate problems with the processing of reward cues, they are less able to make accurate predictions about which kind of behaviors is beneficial for them (Blair, 2010). Reduced reward processing can impair social problem solving, in particular the generation of solutions that are beneficial for them, a topic that may have been underestimated in social information-processing research.

When looking at the brain, the amygdala is thought to be implicated in the formation of stimulus-outcome associations based on environmental feedback and closely interacts with the orbitofrontal cortex, which is implicated in the generation of reinforcement-related expectations (Averbeck & Costa, 2017; Costa & Averbeck, 2020; Rolls, 2004). The orbitofrontal cortex and striatum also play a role in error prediction during learning (Hare et al., 2008; O’Doherty et al., 2006). In a situation where the individual is choosing whether to make a response associated with a particular value, reinforcement expectancy information provided by the striatum on the basis of prior experience is critical (Blair et al., 2018). The striatum and anterior cingulate cortex are also important for prediction of error signals (i.e., detecting a discrepancy between the anticipated and actual outcome). Prediction error signals are thought to facilitate reward and punishment-related feedback learning in terms of error minimization routines. In addition, the ventromedial prefrontal cortex and orbitofrontal cortex represent reinforcement expectancies (Blair et al., 2018; Finger et al., 2011). Children and adolescents with conduct problems have been found to show reduced responses in the orbitofrontal cortex, ventromedial prefrontal cortex, and striatum during both anticipation and response to rewards (Cohn et al., 2015; Finger et al., 2011; Rubia et al., 2009; White et al., 2013).

Difficulties in making correct predictions about which behavior in a certain situation is most beneficial can interfere with children’s and adolescents’ ability to generate situation appropriate solutions. An efficacy study can test the hypothesis whether improving the generation of situation appropriate solutions improves social problem solving and ultimately reduces conduct problems, especially in those children and adolescents who do not show adequate responses in their cognitive repertoire.

In CBT programs, social problem solving is a core theme. Children and adolescents are encouraged to come up with as many solutions as possible which then are categorized into solution types such as help seeking, verbal assertion, compromise, verbal aggression, and physical aggression. One may question whether CBT therapists should work on this with children and adolescents independently of parents and teachers. Children’s learning to generate appropriate solutions is likely to be a slow process, require a lot of practice, and must, therefore also take place in everyday life situations (in vivo practice). The Fast Track study showed that a multiyear preventive intervention offered at schools including the promotion of children’s social-cognitive skills, children’s social skills, and parenting skills, among others, resulted in a decrease of antisocial behavior. This reduction was mediated by its impact on three social-cognitive processes: reducing hostile-attribution biases, increasing the generation of socially competent responses to social problems, and devaluing aggression (Dodge et al., 2013). This study not only demonstrates that increasing children’s generation of appropriate responses to social problems is feasible but is also a mechanism of change and as such constitutes an important aspect of cognitive-behavioral-oriented treatment approaches.

In order to strengthen children’s and adolescents’ ability to produce solutions, CBT therapists may teach parents or foster parents how to assist their child to come up with solutions that are beneficial to the child’s well-being (e.g., solutions resulting in a better relationship with parents, siblings, and peers). This also applies for child care workers in day treatment and residential treatment. Parents can ask their child questions about the social problem such as: ‘What can you do about it?’ And, if needed, parents can give suggestions about which behavior can bring benefits to the child or adolescent himself or herself on the short and long term. But children should also experience for themselves that socially appropriate solutions are rewarding, so that they can become part of their cognitive repertoire.

Therefore, in families where coercive interactions prevail over positive interactions between the child or adolescent and his or her parents and siblings, CBT therapists in their work with parents teach them how to elicit appropriate behaviors in their child by giving positive instructions as well as by relabeling problem behavior in its positive opposite and giving this opposite as an instruction (Kazdin, 2005). If these appropriate behaviors produce a rewarding effect on the child, these behaviors are more likely to re-occur in comparable situations and consequently have a higher likelihood that they become part of the child’s behavioral repertoire for dealing with social situations effectively. As a result, these solutions are stored in long-term memory and are accessible as a possible response to a social situation (i.e., they become part of their cognitive repertoire). Much repetition is needed here before associations between responses and reward are made due to problems in making these associations. CBT therapists may work with teachers in a similar way.

As a complement, collaborative discussions with parents may teach children how to generate mutually satisfactory solutions. This approach, called Collaborative and Proactive Solutions (previously referred to as Collaborative Problem Solving) (Greene, 1998), focuses on helping children and parents learn to proactively and collaboratively solve daily social problems. In a clinical trial in youth with ODD, the Collaborative and Proactive Solutions program was shown to be equivalent to a behavior parent training program (Ollendick et al., 2016).

## Reinforcement-Based Decision-Making

The final step in social problem solving is deciding which alternative will be selected among the ones that have been generated (Crick & Dodge, 1994; Dodge et al., 1986), a step that has often not been included in social information-processing studies. As compared to typically developing boys, school-aged boys with conduct problems who were given the opportunity to select a response among a number of options shown in videos, including prosocial responses, more often selected an aggressive response, and less frequently a prosocial response in situations in which they are being disadvantaged (Matthys et al., 1999). Likewise, adolescents with conduct problems more often selected an aggressive response among the various response options as compared with their typically developing peers in accidental situations (i.e., situations in which they are being disadvantaged by accident) (Van Rest et al., 2020). It is interesting to note that children and adolescents with conduct problems even after an extensive assessment of the social information process in which examples of appropriate responses are shown and numerous questions about the various responses asked, are still inclined to select an aggressive response (Van Rest et al., 2020).

The response-decision process is assumed to be affected by outcome expectations and evaluations based on moral values (Crick & Dodge, 1994). Children and adolescents with aggressive behavior expect aggressive behavior to lead to favorable outcomes (Fontaine et al., 2002; Perry et al., 1986). In addition, children with aggressive behavior have been shown to have positive evaluations of aggressive outcomes (Zelli et al., 1999). Moreover, a stronger belief that aggressive retaliation is acceptable predicts more future aggressive behavior (Zelli et al., 1999).

In neurobiological research, reinforcement-based decision-making studies show that reduced neural responsiveness to reward puts an individual at risk of poor decision making because response choices are less guided by expectations that an action will result in reward relative to punishment (Blair et al., 2018). A meta-analysis of whole-brain fMRI studies showed that the most consistent dysfunction in children and adolescents with conduct problems involves the rostro-dorsomedial, fronto-cingulate, and ventral-striatal regions that mediate reward-based decision making (Alegria et al., 2016). In addition, anterior insular cortex, dorsomedial frontal cortex, and caudate nucleus of the striatum have been found to be implicated in avoidance-related behavior (Blair et al., 2018). Dysfunctions in these regions when making suboptimal choices as a function of expected value have been found in adolescents with conduct problems (White et al., 2014) and are correlated to increased risk for antisocial behavior (White et al., 2016).

Difficulties in decision making based on uncertainties about reward and punishment outcomes can impede children’s and adolescents’ ability to make decisions about appropriate solutions to social problems. An efficacy study can test the hypothesis whether improving decision making based on correct expectations will ultimately result in a reduction of conduct problems, especially in those children and adolescents who have difficulties anticipating that an action will result in a reward or punishment.

In CBT, after children and adolescents have come up with solutions, the therapist asks questions about the consequences of these solutions and about possible alternative solutions in view of making the decision of an appropriate response: ‘What do you think will happen if you do or say that? Will that help solve the problem? What is the direct effect for yourself and for the other? And what is the effect in a week or a month? Is that an appropriate thing to do? Are there other ways to solve the problem?’ Whether just discussing these topics in CBT is sufficient to change children’s and adolescent’s decision-making process remains an open question.

Here, we suggest two approaches that may facilitate the learning processes involved in making appropriate decisions in everyday life situations. First, children and adolescents with conduct problems need to actually experience that appropriate behaviors result in positive consequences. Therefore, therapists in their work with parents, foster parents, and child care workers teach them how to elicit and then reinforce appropriate responses in the child or adolescent. Second, therapists teach parents and other adults how they can assist the child or the adolescent in evaluating various responses and selecting the response for enactment that is most appropriate for him or her not only on the short term (e.g., in terms of reaching a goal for the child himself) but also on the long term (e.g., in terms of positive consequences for the relationship with the other person) (in vivo practice). Parents can learn to ask their child questions such as those previously mentioned. Children and adolescents with conduct problems, however, may have difficulty in making appropriate decisions because response choices are less guided by expectations that an action will result in reward relative to punishment. Therapists, therefore, remind parents, foster parents, and child care workers that much repetition is needed to improve the child’s or adolescent’s decision making. Likewise, CBT therapists may assist teachers in implementing this approach that includes both a behavioral and a cognitive component.

## Response Inhibition

A precondition for using social problem-solving skills is that the tendency to respond impulsively is suppressed. Children with conduct problems are typically action oriented in their social thinking (Lochman & Lampron, 1986; Matthys et al., 1995). So becoming aware that they are encountering a social problem and should think before acting can be a difficult step for them to make in everyday life. In other words, response inhibition or inhibitory control of impulses which is one of the executive functions that regulate people’s thinking and behaviors (Diamond, 2013; Miyake & Friedman, 2012), can be a serious issue for children and adolescents with conduct problems. Impaired response inhibition can prevent them from starting the social problem-solving process and may affect social problem-solving steps such as deciding which response to select among the responses generated (Van Nieuwenhuijzen et al., 2017). While impaired response inhibition should not be expected in children and adolescents with conduct problems as impulsivity is a typical characteristic of Attention-Deficit/Hyperactivity Disorder (ADHD) and not of ODD or CD, the latter disorders and ADHD often co-occur (Angold et al., 1999).

Deficits in response inhibition have not only been found in elementary school children with ADHD, but also in children with conduct problems without comorbid ADHD (Oosterlaan et al., 1998). Similarly, impairments in inhibition were observed for both preschool children with ADHD and preschool children with conduct problems with and without ADHD comorbidity relative to typically developing children (Schoemaker et al., 2012). In contrast, adolescents with ADHD, conduct problems, and autism spectrum disorder show a high number of failed inhibitions on a Go/NoGo task relative to typically developing children. However, post hoc analyses suggest that ADHD symptoms may be in part driving the increased level of unsuccessfully inhibited no-go trials in the conduct problems group (Leno et al, 2018). Even if this would be the case, given the co-occurrence of ADHD symptoms and conduct problems at a greater than random rate (Waschbusch, 2002), impaired response inhibition is still relevant for the conduct problems group (see systematic review and meta-analysis by Bonham et al., 2021). In addition, when motivational factors such as reward and punishment are included in response inhibition tasks (e.g., the response perseveration task), impairments in these so-called ‘hot executive functions’ seem to be clearly associated with conduct problems (Matthys et al., 1998, 2004), and even more than with ADHD (Van Goozen et al., 2004). For reviews of the neuroimaging literature, see Rubia (2011) and Noordermeer et al. (2016).

In a review of fMRI studies, Blair et al. (2018) conclude that studies examining different paradigms involving response inhibition (as a ‘cold executive function’) report no differences in recruitment of regions implicated in response control, including the inferior frontal gyrus, anterior insular cortex, and dorsomedial frontal cortex, between children and adolescents with conduct problems and controls. The authors note that several of these studies excluded youth with conduct problems with comorbid ADHD. A study that did not control for the presence of ADHD showed reduced anterior insular activity on a cognitive interference (Stroop) task. The extent of impairment did not particularly relate to severity of conduct problems but did positively correlate to ADHD symptom severity (Hwang et al., 2016). In our opinion, this does not make the finding any less relevant. As ADHD often is associated with conduct problems (Angold et al., 1999), impaired response inhibition may be a characteristic of the child or adolescent referred for the treatment of conduct problems. In sum, response inhibition may be impaired in children and adolescents with conduct problems either due to the association with ADHD (symptoms) or to motivational demands (reward and punishment) included.

With regard to social problem solving, impaired response inhibition can prevent children and adolescents with conduct problems from starting the thinking process before acting, especially those with either comorbid ADHD or associated ADHD symptoms. Impaired response inhibition may also affect these children’s and adolescents’ social problem solving such as generating solutions and making decisions. An efficacy study can test the hypothesis whether enhancing response inhibition through training programs (see further Kofler et al., 2018, 2020) affects social problem solving in everyday life and ultimately results in a reduction of conduct problems, especially in those children and adolescents with either comorbid ADHD or associated ADHD symptoms. Likewise, an efficacy study can test the hypothesis whether enhancing response inhibition by psychostimulants in children and adolescents with conduct problems and ADHD comorbidity affects social problem solving in everyday life and ultimately results in a reduction of conduct problems.

In CBT, thinking prior to acting is a theme. In a study of the child component of Coping Power, effectiveness of group delivery was compared to individual delivery. According to teachers, children with low levels of inhibitory control appeared to profit more from individual delivery of Coping Power than children with high levels of inhibitory control, suggesting that individual delivery offers opportunities for tailoring CBT to children’s individual needs (Lochman et al., 2015).

Although thinking prior to acting is addressed by CBT, the issue of starting the thinking process when children and adolescents are facing a social problem in everyday life at home and at school probably may need more attention. We, therefore, suggest that CBT therapists instruct parents, foster parents, child care workers, and teachers to assist the child or adolescent in withstanding his or her impulsive urges by engaging into the thinking process of social problem solving that children and adolescents learn in CBT during daily life (in vivo practice). When children or adolescents ask for help when they face a social problem, parents and teachers assist the child or adolescent if necessary by asking questions like: ‘What is the problem? What are some solutions?’ etc. Likewise, when parents and teachers see the problem arise before their eyes, they can ask those questions. It is, however, complicated when the problem occurs out of sight of adults. It may help to discuss with the child and adolescent in CBT the type of social situation he or she finds particularly difficult to deal with. For example, when the child finds out that he or she has been left out of a group, game, or activity of peers or when a peer performs better than the child in a game (Dodge et al., 1985; Matthys et al., 2001). When children and adolescents know which social situation is problematic for them, this awareness can help them to engage in the thinking process and ask themselves questions on how to solve the problem.

In line with a conceptual framework for combined neurocognitive and skill-based treatment approaches for youth with ADHD (Chacko et al., 2014), we suggest that executive function training programs are included in the treatment of children and adolescents with conduct problems and poor response inhibition associated with ADHD. While training programs targeting working memory and response inhibition have been less successful than initially anticipated, results of recent studies on central executive training targeting working memory, with effects on response inhibition and hyperactivity, are promising (Kofler et al., 2018, 2020). In a review of interventions and approaches for improving executive functions, Diamond and Ling (2016) suggest that while challenging of executive functions is necessary for improving them, benefits of improving executive functions will be greater if children’s and adolescents’ emotional, social, and physical needs are also addressed. For instance, stress, sadness, loneliness, not enough sleep, and lack of physical activity have a negative impact on executive functioning (Diamond & Ling, 2016). Parents can, therefore, create conditions (e.g., healthy life style) for their child to make optimal use of their executive functions among which response inhibition.

## Affective Empathy

Empathy is the understanding of another’s emotional state (cognitive empathy) as well as the ability of sharing/feeling another’s emotional state (affective empathy). The cognitive aspect involves finding out or inferring and understanding another person's emotional state (e.g., sadness, fear, or pain). The affective aspect includes the emotional response to ourselves in perceiving the other person's feeling. In particular, this emotional response is appropriate to or congruent with another’s situation than to one’s own (Eisenberg & Fabes, 1990; Hoffman, 1984; Moul et al., 2018). Empathy is associated with prosocial behavior such as helping and comforting others and contributes to the inhibition of antisocial and aggressive behavior (Eisenberg & Miller, 1987; Miller & Eisenberg, 1988). However, systematic and meta-analytic reviews of empathy show considerable inconsistencies in the assumed association between empathy and conduct problems. Differences in the measures used and the conceptualization of empathy have been suggested to, at least in part, contribute to the heterogeneous findings (Moul et al., 2018).

Lack of empathy is one of cardinal features of callous-unemotional (CU) traits (Frick et al., 2014). In the DSM-5, CU traits are labeled as ‘limited prosocial emotions,’ a diagnostic specifier for individuals who meet full criteria for Conduct Disorder. Limited prosocial emotions include the following characteristics: lack of empathy, lack of remorse or guilt, shallow or deficient effect, and unconcerned about performance (American Psychiatric Association, 2013). Children and adolescents with both severe conduct problems and elevated CU traits are at risk for more severe and persistent antisocial outcomes (Frick et al., 2014). They also tend to be less responsive to psychological treatment (Frick et al., 2014).

Blair (1995) suggested that in humans, a victim’s pain and distress induce similar feelings of distress in the aggressor, which in turn stops further aggressive behavior. Children and adolescents with a deficit in this mechanism will be less likely to learn to avoid harming other individuals, because the distress of other individuals is less aversive for them. These children and adolescents are, therefore, more likely to continue displaying behaviors that harm others to achieve their goals.

Distress-related cues, particularly fearful expressions, play an important role in inhibiting antisocial behavior (Blair, 2001). A meta-analysis showed a strong association between antisocial behavior and deficits in recognizing fearful expressions (Marsh & Blair, 2008). Consistent with this, in an fMRI study, children and adolescents with conduct problems and CU traits showed reduced amygdala responsiveness during the presentation of fearful facial expressions in comparison to healthy controls and youth with ADHD. Interestingly, functional connectivity analyses demonstrated lower correlations between the amygdala and ventromedial prefrontal cortex in youth with conduct problems and CU traits as compared to healthy controls and youth with ADHD (Marsh et al., 2008). Impairments in amygdala-ventromedial prefrontal cortex connectivity are suggested to be associated with antisocial behavior as a result of instrumental behavior that is inappropriately modulated by others’ distress (Marsh et al., 2008). In addition, adolescents with conduct problems and psychopathic traits, including reduced empathy and guilt, showed reduced activity in the rostral anterior cingulate cortex, ventral striatum, and amygdala in response to observing increased pain in others (Marsh et al., 2013). Also, reduced activity in the insula while viewing others being harmed was related to children’s greater CD symptoms and CU traits (Michalska et al., 2016). Interestingly, reduced left anterior insula and inferior frontal gyrus responses in adolescents with conduct problems and CU traits relative to normal controls were found when participants were asked to judge their *own* emotional reactions to fearful and angry expressions (Klapwijk et al., 2016). These findings suggest that adolescents with conduct problems and CU traits resonate less with the feelings of others (Klapwijk et al., 2016). Impairments in affective empathy have also been demonstrated in children and adolescents with conduct problems without CU traits (Martin-Key et al., 2017) and in children aged 7–11 years with disruptive behavior referred into a crime prevention program (Hunnikin et al., 2020).

Difficulties in affective empathy in response to other’s distress can result in the maintenance of aggressive behavior. An efficacy study can test the hypothesis whether improving affective empathy, in particular in response to other’s distress, using virtual reality (see further Dellazizzo et al., 2019), affects aggressive behavior, especially in those children and adolescents with limited prosocial emotions.

Currently, in CBT, much attention is given to perspective taking, in particular understanding another person’s intentions as children and adolescents with aggressive behavior are inclined to attribute hostile intentions to others (De Castro et al., 2002; Verhoef et al., 2019). Attention is also given to better understand the emotions of others, but whereas cognitive empathy is a central theme, affective empathy is not. Here, we suggest that improving affective empathetic responding, in particular in response to other’s distress, should be a target in CBT. Children and adolescents with conduct problems, especially those with limited prosocial emotions, must learn to pay attention to the child’s distress towards whom they start displaying aggressive behavior and must experience themselves how it feels like if this is done to them, with a view to stopping this behavior. This requires a lot of practice, possibly adding virtual reality, as individuals tend to respond realistically to virtual simulations of real-life events (Dellazizzo et al., 2019), to the everyday life situations. Parents, foster parents, child care workers in day treatment and residential treatment centers, and teachers can learn how to help the child or adolescent in paying attention to peer’s distress when the child or adolescent starts showing behavior that physically and/or mentally harms others (in vivo practice).

Lack of remorse or guilt is another characteristic of limited prosocial emotions, related to lack of empathy. The amygdala plays a role in care-based moral judgements (Blair, 2007). As already discussed, reduced amygdala responsiveness to the distress of other individuals has been shown in children and adolescents with conduct problems and with CU traits (Marsh et al., 2008). In line with this, adolescents with conduct problems and psychopathic traits showed reduced amygdala activity when making judgements about legal/illegal actions; thus, psychopathic traits appear to be associated with these adolescents’ ability to attach the appropriate valence to actions of varying moral permissibility (Marsh et al., 2011). These findings suggest that for adolescents with conduct problems and limited prosocial emotions, it is appropriate to include moral reasoning in CBT (see Aggression Replacement Training; Goldstein et al., 1998), for example in the context of generating solutions to social problems. A slow learning process must be taken into account here as well.

## Discussion

The mean effect size of psychotherapy for conduct problems in children and adolescents has been shown to decrease over the last 50 years (1963–2016), suggesting that adjustments are needed in some of the approaches that have been followed thus far (Weisz et al., 2019). Most evidence-based CBT programs were developed during the last three decades of the previous century and only slightly updated in the present century. Results of neuroimaging research into a series of psychological functions have not been incorporated in this update: anger recognition, the ability to generate appropriate solutions to social problems, reinforcement-based decision making, response inhibition, and affective empathy. We propose that these psychological functions deserve more attention in CBT and that working on these psychological abilities only during CBT sessions is not sufficient. Children’s and adolescents’ use of these psychological abilities in everyday life, on the other hand, must be elicited, supported, and reinforced by parents, foster parents, or child care workers in day treatment and residential centers. Therefore, parents or other adults must be intensively involved in the CBT of their child, by participating in part of the sessions, but especially by finding out, with the help of the therapist, how the learning processes of which the first steps were taken in the sessions can be continued in everyday life.

In addition, the increased child’s and adolescent’s competence to use cognitive skills goes hand in hand with learning behavioral skills. For example, a prerequisite for generating an appropriate solution or for selecting an appropriate one between different solutions generated is that children and adolescents have experienced that appropriate solutions work for them and as a result become part of their behavioral repertoire. Thus, therapists and parents, foster parents and child care workers in day treatment and residential treatment centers, need also to work on the improvement of the child’s or adolescent’s behavioral repertoire using the typical parenting skills targeted in behavioral parent training such as giving positive instructions and praising. If these appropriate behaviors work for the child and the adolescent and are perceived as rewarding by them, they are stored in long-term memory, can be generated as possible responses, and finally chosen as the best response based on positive outcomes both on the short and long term. Much perseverance is needed here before associations between responses and reward are made due to problems in making these associations.

The developing brain of children and adolescents is plastic and exhibits greater learning capacity as compared to the adult brain. It is, therefore, not unreasonable to assume that psychological-based interventions at a relatively early age have a positive and lasting effect on brain maturation and neural organization (Ismael et al., 2017). There is, for instance, evidence that experience-based brain plasticity can have positive effects paralleled by associated functional as well as structural neuroanatomical changes in children with ADHD (Hoekzema et al., 2011). In addition to making use of these developmental windows of opportunity by promoting learning during CBT sessions as well as in the everyday life setting, promoting a healthy life style is likely to have positive effects as well (Diamond & Ling, 2016). For example, a balanced eating diet, sufficient amount of sleep, and physical activity (e.g., sports) may improve learning and executive functioning which, in turn, enhance psychological functions associated with anger regulation and social problem solving.

Just as children and adolescents with conduct problems differ in symptoms (e.g., reactive aggression and CU traits), so they differ in the psychological functions that need to be specifically addressed, such as anger recognition, generating appropriate solutions, or affective empathic responding. Therefore, CBT should be tailored to the individual psychological disabilities. In addition to working with the child or adolescent, engaging parents or other adults provide an opportunity for the therapist to personalize CBT. A modular approach as first used in CBT for anxiety disorders and subsequently for other types of psychopathology may be appropriate here (Chorpita et al., 2004; Evans et al., 2020). A modular approach preserves the benefits of standardization of manualized protocols, while, at the same time, modules can be flexibly arranged so that the content, order, and dose are adjusted to the child and adolescent characteristics. If deemed appropriate, the use of individual or group sessions can also be included in this approach.

Group format is engaging for children and adolescents and offers many opportunities for modeling. However, group formats can sometimes be unnecessarily long for individual children or adolescents if they already display specific anger management or social problem-solving skills in their repertoire. If this is the case, then the number of group sessions with the child or adolescent can be reduced to make room for individual sessions tailored to the individual characteristics of the child’s or adolescent’s psychological functions that need improvement.

For example, psychoeducational group sessions are aimed at introducing the set of anger management and social problem-solving skills that will be worked on in individual sessions later. Thus, children and adolescents first become familiar with the cognitive and behavioral skills during group psychoeducational sessions on anger management (one module) and social problem solving (another module). Subsequently, in individual sessions in which the parents or other adults (e.g., child care workers) and the child or adolescent participate, parents learn to elicit, support, and reinforce their child’s use of anger management skills (several modules) and social problem-solving skills (several modules) during everyday life. In these individual sessions, working on the psychological functions is tailored to the individual child’s characteristic impaired functions. Homework assignments are used to practice skills tailored to the individual child, adolescent, and parent at home. With regard to effectiveness of individual versus group delivery of CBT, according to parents, individual delivery of the child component of Coping Power was as effective as group delivery, whereas according to teachers, individual delivery was more effective (Lochman et al., 2015).

For the improvement of anger management and social problem-solving skills, a lot of practice is needed. The school offers unique opportunities for this. Indicated preventive interventions that also target children with clinical levels of conduct problems, have chosen the school as the social context for intervention, also for practical reasons. The Fast Track project has shown that the intervention’s impact on the prevention of later crime and to a lesser extent of general and mental health problems can be accounted for by improvements in self-regulation and problem-solving skills (Sorenson et al., 2016). Children and adolescents spend a lot of time at school in a wide variety of social contexts that may be a problem for them, such as situations in which the child is being disadvantaged or must cope with competition (Dodge et al., 1985; Matthys et al., 2001). Although the learning processes aimed at anger regulation and social problem solving are initiated in the CBT sessions, they must be given a chance to continue at school. Therefore, the CBT therapist shares crucial learning topics for the student with the teacher with a view to creating learning opportunities for the student. Just like parents, teachers may also have collaborative discussions with students how to proactively and collaboratively solve daily social problems (Greene, 1998; Ollendick et al., 2016).

Engaging families in psychological treatment for conduct problems is challenging (Acri et al., 2018). Data from 262 studies for example showed that at least 25% of the parents of children aged 2–12 years identified as appropriate for behavioral parent training do not start treatment, and an additional 26% begin, but drop out before completing treatment (Chacko et al., 2016). When behavioral parent training is the only psychological therapy suggested by the clinician who performed the clinical evaluation of the child or adolescent, parents may not start treatment because they think that their parenting skills are of a sufficient level and their child has to deal with his or her problems. These parents may be right as it has been shown that not all families benefit equally from behavioral parent training, in part because parenting skills in some families are not clearly inappropriate (Van Aar et al., 2019). Also, parents may drop out of treatment after a few sessions because they think their parenting skills have now reached an adequate level and the child now needs to work on his or her problems. So involving the child himself or herself in the treatment either from the start or after a series of behavioral parent training sessions in order to improve his or her anger regulation and social problem-solving skills may increase the likelihood that the treatment will start or be completed.

There is consensus among clinical researchers that children derive increasing benefit from CBT with increasing age (Fairchild et al., 2019). Adding CBT to behavioral parent training may increase the effect of behavioral parent training in children aged from 7 years on. Kazdin and colleagues examined whether the combination of Problem-Solving Skills Training (a CBT program) and Parent Management Training (a behavioral parent training program) generated an intervention that was more potent than either treatment alone in children with conduct problems aged 7–13 years (Kazdin et al., 1992). The combined treatment led to more marked changes in antisocial behavior of the child and in parental stress than Problem-Solving Skills Training only and Parent Management Training only. The effect was also evident in the proportion of children that the combined treatment placed within the normative range of functioning in comparison with either treatment alone (Kazdin et al., 1992).

For the treatment of adolescents with conduct problems, CBT can be added to family-based psychotherapy (e.g., Functional Family Therapy; Alexander et al., 2013) or may become part of interventions targeting multiple systems (e.g., Multisystemic Therapy; Henggeler et al., 2009). Increasing the effectiveness of CBT for adolescents is needed as a meta-analysis of Multisystemic Therapy, specifically indicated for adolescents with the most severe conduct problems such as violent offenders and conduct problems associated with substance abuse, showed small effect sizes: *d* = 0.20 for delinquency and *d* = 0.29 for psychopathology (Van der Stouwe et al., 2014). Likewise, as already mentioned, the ES of CBT in youths treated for antisocial behavior in residential settings is only *d* = 0.25 (Armelius & Andreassen, 2007).

Finally, the perspective we adopted was a non-systematic review of the available neuroimaging literature to examine the neural basis of psychological functions that are implicated in the effectiveness of CBT programs. However, we acknowledge that the number of neuroimaging studies is limited. In addition to the five psychological functions discussed in the present review, other psychological functions, such as working memory, which are not included in the CBT programs may be relevant as well.

## Conclusion

Although the literature on anger regulation and social problem solving is extensive, the present review of functional neuroimaging studies suggests that some psychological functions targeted in CBT may need more attention: anger recognition, the ability to generate situation appropriate solutions to social problems, reinforcement-based decision making, response inhibition, and affective empathy. Directly following from the observations that these psychological functions may be impaired in conduct problems, a number of suggestions to increase therapeutic effectiveness of CBT were made. While these require a close collaboration of the therapist with parents, teachers and child care workers in day treatment and residential centers, taking into consideration specific psychological dysfunctions may be beneficial to improving the effectiveness of CBT in the treatment of conduct problems.
